# Identification of Marker Peptides in Gelatins from Sika Deer (*Cervus nippon*) Using Ultra-High-Performance Liquid Chromatography–Quadrupole-Exactive-Orbitrap Mass Spectrometry

**DOI:** 10.3390/molecules30071528

**Published:** 2025-03-29

**Authors:** Kouharu Otsuki, Aya Nomizo, Mi Zhang, Dongxia Li, Takashi Kikuchi, Wei Li

**Affiliations:** 1 Faculty of Pharmaceutical Sciences, Toho University, Miyama 2-2-1, Funabashi 274-8510, Japantakashi.kikuchi@phar.toho-u.ac.jp (T.K.); 2 Department of Medical Laboratory, Medical College of Dalian University, Dalian 116622, China

**Keywords:** sika deer, gelatin, marker peptide, LC-MS/MS

## Abstract

Gelatin from deer has garnered attention as a high-value health-promoting resource given its history of usage as a traditional Chinese medicine and recent studies demonstrating its biological activities. Mass spectrometry-based methods have increasingly been employed for species identification in collagen-based materials, effectively addressing challenges in quality control and authenticity verification. This study aims to identify characteristic marker peptides in gelatins from sika deer (*Cervus nippon*) to support their effective use as a health-promoting resource. Gelatin samples were enzymatically digested, and the resulting peptide mixtures were analyzed using ultra-high-performance liquid chromatography coupled with quadrupole Q-Exactive-Orbitrap mass spectrometry (UHPLC-Q-Exactive-Orbitrap MS). Marker peptide candidates were selected based on their high detection intensity and a literature review. Among the 28 selected marker peptide candidates, four peptides (P11, R2, R3, and R4) were defined as characteristic of sika deer gelatin. Comparative analyses with gelatins derived from donkey hide, bovine, porcine, and fish samples further confirmed the specificity of these peptides. These findings establish a robust analytical method for verifying the authenticity of sika deer gelatin, contributing to its safe and effective use as a health-promoting resource.

## 1. Introduction

Wild deer populations have surged globally for decades since reduced hunting, the absence of natural predators, habitat modifications induced by agricultural and forestry, as well as climate change, have posed significant ecological and socio-economic challenges [1]. Sika deer (*Cervus nippon*), a native ungulate species in Japan, has reached ecologically overabundant levels, resulting in substantial damage to agriculture and forestry, disruptions to ecosystems, economic losses, and increased risks of zoonotic disease transmission [2,3]. To address these challenges, Japan has implemented regular hunting and promoted the use of harvested deer, primarily for meat production [4,5]. However, the alternative applications of sika deer byproducts, particularly in the health, pharmaceutical, or industrial fields, remain underdeveloped and require further investigation [6].

Deer have been integral to traditional Chinese medicine for centuries, with antlers, tails, and hides utilized for their purported therapeutic benefits [7]. Among these, deer antlers are particularly valued for their tonic properties and potential to alleviate aging-related symptoms. Recently, deer antlers have garnered attention as a renewable resource, with studies highlighting their antioxidant, anti-inflammatory, neuroprotective, and antitumor effects [8,9,10]. Several studies on sika deer have demonstrated that antler extracts exhibit anticancer and nephroprotective effects in both in vitro and in vivo models [11,12,13]. In addition, among the different parts of sika deer antlers, wax slices have been reported to possess the most significant immunological activity and anti-fatigue effects [14]. Gelatin derived from deer antlers was traditionally used to treat symptoms such as blood deficiency-related dizziness, joint soreness, and fatigue-induced emaciation. It has also attracted attention in recent years for its high value as a health-promoting resource [15]. Research on the biological activities of deer antler gelatin has demonstrated its antioxidant, anti-inflammatory, immunomodulatory, and anti-dementia potential due to protecting neuronal mitochondria from oxidative damage [16,17,18,19]. Additionally, peptides derived from deer antler gelatin have demonstrated protective effects against lung and liver injuries, further emphasizing its therapeutic potential [20,21]. In our previous research, as part of an initiative to enhance the use of sika deer as a health-promoting resource, we demonstrated that gelatin from sika deer antlers, hides, and bones exhibits a notable in vitro antioxidant activity, comparable to that of gelatin from donkey hide, a well-known traditional Chinese medicine (Ejiao). Both deer antler and donkey hide gelatins predominantly consist of peptides and proteins derived from collagen, a biopolymer highly valued for its functional and technical properties across the food, pharmaceutical, and cosmetic industries [10,22,23].

Mass spectrometry (MS) is a widely utilized analytical technique known for its exceptional sensitivity and selectivity, enabling rapid, high-throughput screening to detect metabolic markers in complex biological samples. Recently, MS-based approaches have been increasingly utilized for species identification in various animal-derived products, such as adhesives [24,25], leather [26], bone [27], meat [28,29], and gelatin [30,31]. These techniques have also been applied to identify species in animal-derived medicinal products, including deer and donkey gelatins [32,33,34,35,36,37]. Collagen-based materials are widely derived from various animals, however, the diversity of collagen sources presents significant challenges for quality control, particularly in ensuring species-specific authenticity. The issue is especially critical in applications such as traditional medicines and functional health supplements, where species identification is essential not only for therapeutic efficacy but also for religious and health considerations [32,33,38]. Despite the increasing demand for reliable species-specific identification methods, research focusing on sika deer remains limited.

In this work, we aim to identify characteristic marker peptides in gelatins from sika deer using qualitative analysis with liquid chromatography–mass spectrometry (LC-MS). In addition to sika deer gelatin, we analyzed high-resolution mass spectrometry data from trypsin-digested peptide mixtures of commercially available gelatins and utilized the precise sequence information of type I collagen-derived peptides identified through MS/MS fragmentation patterns to distinguish the gelatin origins. This approach will facilitate the differentiation of sika deer gelatin from other animal-derived gelatins, ensuring its safe, effective, and authentic use as a health-promoting resource.

## 2. Results and Discussion

### 2.1. Selection of Marker Peptide Candidates

Target proteins are typically enzymatically digested into peptide fragments using trypsin, and mass spectrometry (MS) is highly effective in simultaneously detecting these peptides with high sensitivity, allowing for species identification based on interspecies differences in amino acid sequences. Gelatins from the antlers, hides, and bones of sika deer were hydrolyzed with trypsin, and the resulting peptide mixtures were analyzed using ultra-high-performance liquid chromatography coupled with quadrupole Q-Exactive-Orbitrap mass spectrometry (UHPLC-Q-Exactive-Orbitrap MS). High-resolution mass spectrometry data of enzymatically digested peptides from all sika deer gelatin samples were obtained (Figure S1). Using PeptideShaker software (version 3.0.8) [39], 134 peptides from antler gelatin, 157 peptides from hide gelatin, and 172 peptides from bone gelatin were identified (Tables S1–S3).

To identify marker peptides for distinguishing gelatin from sika deer, 28 marker peptide candidates were first selected based on the following criteria (Table 1). Peptides readily detectable in sika deer gelatin were chosen as marker candidates (P1–P12) if they met the following conditions: (1) Peptides with relative intensities > 50% in the high-resolution mass spectrometry data of antler, hide, and bone gelatin. (2) Peptides identified with 100% confidence using PeptideShaker software. Using these conditions, 12 candidate peptides (P1–P12) were identified, with 9 originating from the type I collagen α1 chain (P1, P3–P5, P7, P8, and P10–P12) and 3 from the type I collagen α2 chain (P2, P6, and P9). Additionally, peptides previously reported in the literature as deer-specific markers (R1–R16) were also chosen as marker candidates in this study [26,30,32,33,34,36,39]. Among these, 4 peptides (R1, R7, R8, and R16) originated from the α1(I) chain, while 13 peptides (R2–R6 and R9–R15) were derived from the α2(I) chain.

### 2.2. Definition of Marker Peptide of Gelatins from Sika Deer

**Figure 1:**
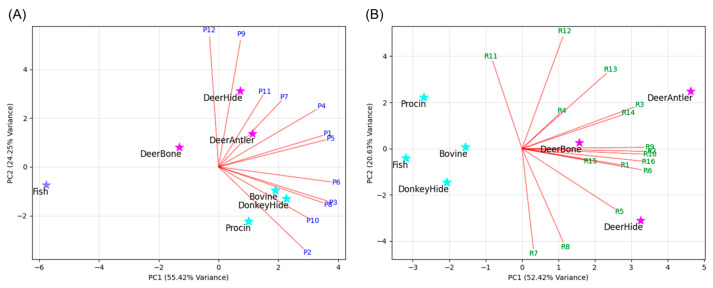
Principal component analysis (PCA) of sika deer gelatin and commercially available gelatins with peptides (**A**) P1–P12 and (**B**) R1–R16.

Subsequently, gelatins commonly used in the pharmaceutical, food, and cosmetic industries, including donkey hide, bovine, porcine, and fish gelatin, were enzymatically digested, and high-resolution mass spectrometry data of the resulting peptide mixtures were obtained, following the same protocol as for sika deer gelatin. To verify the specificity of marker peptides for sika deer gelatin in the broader context of commonly used animal-derived gelatins, a comparative analysis was performed between the high-resolution mass spectrometry data and sequence information from public databases of the other animal species and the analytical data obtained for sika deer gelatin. To evaluate the presence of selected marker peptide candidates in all gelatin samples, the relative abundance of each candidate was calculated based on the peak area, using the trypsin-derived peptide peak (LGEHNIDVLEGNEQFINAAK, *m*/*z* 737.71, triple-charged), consistently detected across all samples, as a reference (Table 2). Due to significant differences in detection intensity between peptides P1–P12 and R1–R16, principal component analysis (PCA) was performed separately for the peptide P group and R group. As shown in the biplot (Figure 1), among the peptide P group, P1, P4, P5, P7, P9, P11, and P12 contributed to the identification of sika deer gelatin. Similarly, among the peptide R group, R1–R6, R9, R10, and R13–R16 were found to contribute to the identification of sika deer gelatin. Furthermore, to ensure the clear identification of sika deer gelatin, marker peptides were defined based on the following criteria. For P1–P12, peptides must show high detection intensity in all sika deer gelatin samples (peak area ratio relative to the trypsin-derived peptide > 3.0) and not be detected in at least two other animal-derived gelatin samples (peak area ratio < 1.0). For R1–R16, peptides must be detected in all sika deer gelatin samples (peak area ratio > 0.5) and not detected in at least two other animal-derived gelatin samples (peak area ratio < 0.1).

Although commercial gelatin samples from horses, sheep, and goats (closely related ungulates to deer) were unavailable, the amino acid sequences of type I collagen α1 and α2 chains from these closely related ungulates were obtained from the UniProt database. Basic Local Alignment Search Tool (BLAST+, v2.16.0) searches were conducted to verify the uniqueness of the selected marker peptide candidates (Table 2). To enhance specificity, peptides with identical sequences across all three ungulates were excluded as marker peptides. As a result, among the 28 marker peptide candidates, P11, R2, R3, and R4 were defined as both specific to sika deer gelatin and exhibiting relatively high detection intensities. The combination of multiple marker peptides has been shown to improve the reliability and accuracy of species identification [25,26]. In light of this, the selected set of four marker peptides is considered well suited for distinguishing and verifying the use of gelatin from sika deer.

### 2.3. Interpretation of Product Ion Spectra for Defined Marker Peptides

**Figure 2:**
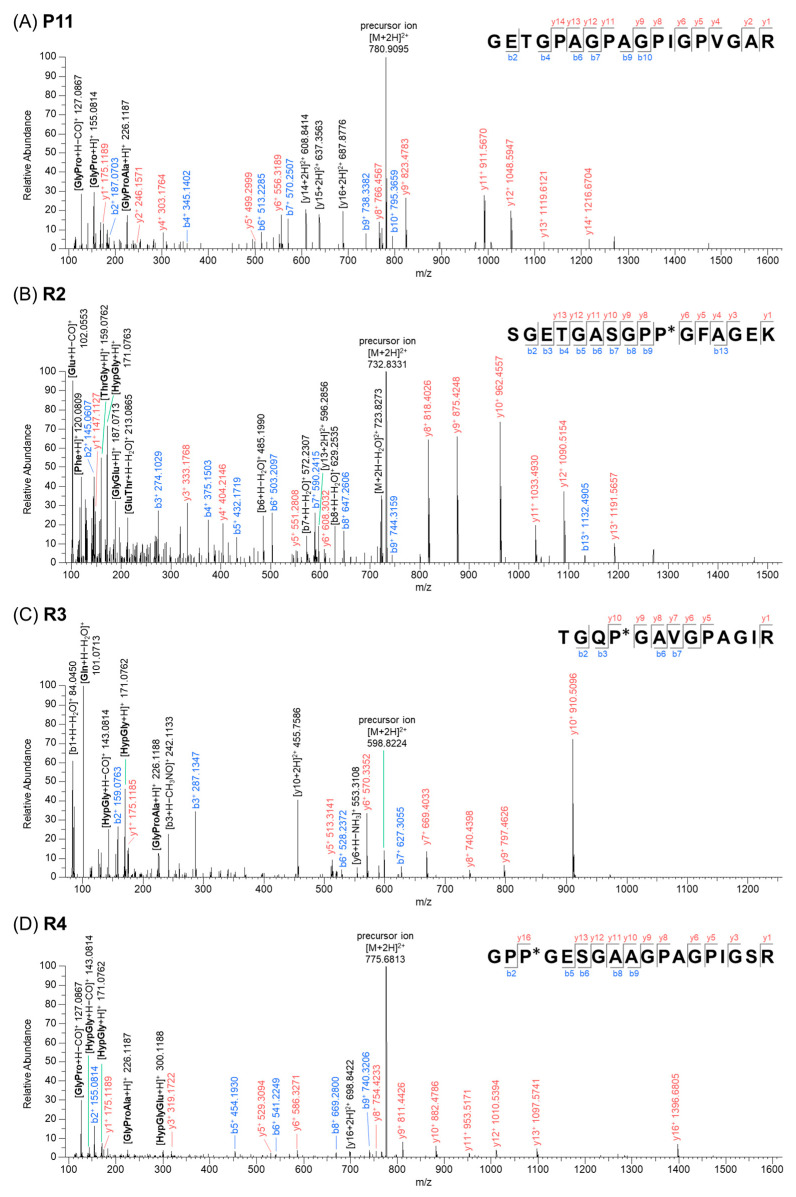
Product ion spectrum of marker peptides obtained from ESI-MS/MS (P* and Hyp indicate hydroxyproline). (**A**) Peptide P11, (**B**) peptide R2, (**C**) peptide R3, and (**D**) peptide R4.

The mass spectrum and product ion spectrum for the marker peptides P11, R2, R3, and R4 detected in sika deer gelatin are shown in Figures S3–S6. In electrospray ionization mass spectrometry (ESI-MS), b-ions and y-ions were predominantly generated. In addition to these ions, high-intensity product ions were observed, and their compositions were estimated based on accurate mass data and to examine whether the identified amino acid sequence was reasonable (Figure 2). The precursor ion of P11 was identified as *m/z* 780.91 (double-charged). The sequence GETGPAGPAGPIGPVGAR was confirmed based on the y-ion series of *m/z* 175.12 (y1^+^), 246.16 (y2^+^), 303.18 (y4^+^), 499.2999 (y5^+^), 556.3189 (y6^+^), 766.4567 (y8^+^), 823.4783 (y9^+^), 911.57 (y11^+^), 1048.59 (y12^+^), 1119.61 (y13^+^), 1216.67 (y14^+^) as well as b-ion series *m*/*z* 187.07 (b2^+^), 345.14 (b4^+^), 513.23 (b6^+^), 570.25 (b7^+^), 738.34 (b9^+^), and 795.37 (b10^+^). The sequence could be found in COL1A1 from white-tailed deer (UniProt Accession No. A0A6J0Z7P5, aa976–aa993) and bovine (*Bos taurus*) (UniProt Accession No. P02453, aa11066–aa1083). Furthermore, the fragmentation of y15^+^ or y12^+^ resulted in generated characteristic product ions at *m/z* 127.09 [GlyPro − CO (carbon monoxide) + H]^+^, 155.08 [GlyPro + H]^+^, and 226.12 [GlyProAla + H]^+^, while product ions at *m/z* 608.84, 637.36, and 687.88 were identified as double-charged ions derived from cleavage at the y14, y15, and y16 positions.

The amino acid sequences identified from the analysis of the y-ions and b-ions of the peptides R2, R3, and R4 were consistent with the sequences reported in the literature [26,32,37]. These results were further validated through detailed MS/MS fragmentation analysis. Namely, for the peptide R2, in addition to the product ions associated with b-ions and y-ions, specific product ions derived from individual amino acids were observed at *m/z* 102.05 [Glu − CO + H]^+^, 120.08 [Phe + H]^+^, 159.08 [ThrGly + H]^+^, 171.08 [HypGly + H]^+^, 187.07 [GlyGlu + H]^+^, and 213.09 [GluThr − H_2_O + H]^+^. Similarly, for the peptide R3, characteristic product ions were observed at *m/z* 101.07 [Gln − H_2_O + H]^+^, 171.08 [HypGly + H]^+^, and 226.12 [GlyProAla + H]^+^, while for the peptide R4, distinctive product ions were observed at *m/z* 127.09 [GlyPro − CO + H]^+^, 143.08 [HypGly − CO + H]^+^, 171.08 [HypGly + H]^+^, 226.12 [GlyProAla + H]^+^, and 300.12 [HypGlyGlu + H]^+^. The analysis of these product ions revealed amino acid combinations consistent with the identified sequences of the peptides R2, R3, and R4, further substantiating their accurate characterization.

### 2.4. Identification of Gelatin from Sika Deer by Defined Marker Peptides

**Figure 3:**
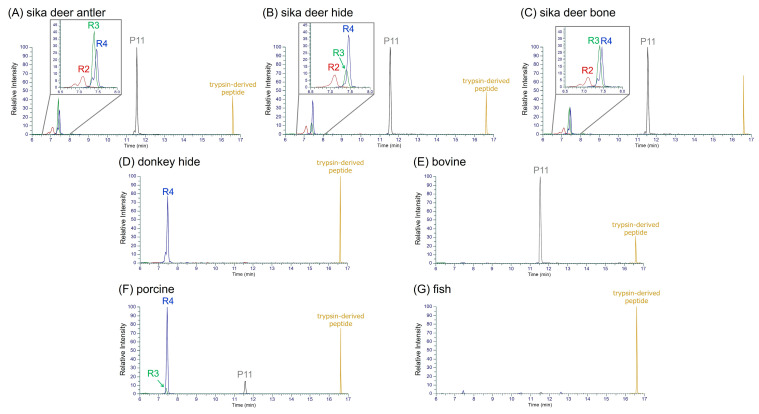
Extracted ion chromatograms (XICs) of marker peptides in sika deer gelatin and commercially available gelatins. (**A**) Sika deer antler, (**B**) sika deer hide, (**C**) sika deer bone, (**D**) donkey hide, (**E**) bovine, (**F**) porcine, and (**G**) fish.

The occurrence patterns of the selected marker peptides across various gelatin samples were compared using extracted ion chromatograms (XICs) of their precursor ions (Figure 3). All four marker peptides were detected in sika deer gelatin, whereas only specific marker peptides were detected in gelatin from other animals. Among the three kinds of sika deer gelatin (antler, hide, and bone), the peptide P11 consistently exhibited the highest intensity. The peak areas of R2, R3, and R4 varied depending on the gelatin source, showing distinct patterns: R4 > R3 > R2 for antler gelatin (Figure 3A), R4 > R3 = R2 for hide gelatin (Figure 3B), and R4 = R3 > R2 for bone gelatin (Figure 3C). In contrast, the peptide R4 was strongly detected in donkey hide and porcine gelatins (Figure 3D,F), while P11 was prominent in bovine gelatin (Figure 3E), and no marker peptides were detected in fish gelatin (Figure 3G). These findings demonstrate that combining multiple marker peptides effectively distinguishes sika deer gelatin from other animal-derived gelatins. Furthermore, the differences in the peak area values of the selected marker peptides suggest the potential to distinguish the anatomical origin of the gelatin within sika deer samples.

## 3. Materials and Methods

### 3.1. Chemicals and Materials

Trypsin, from porcine pancreas (4400 USP units/mg, for biochemistry), and ammonium bicarbonate (for proteomics) were purchased from FUJIFILM Wako Pure Chemical Co. (Osaka, Japan). LC-MS grade distilled water containing 0.1% (*v*/*v*) formic acid and acetonitrile containing 0.1% (*v*/*v*) formic acid were obtained from Kanto Chemical Co., Inc. (Tokyo, Japan). Three kinds of sika deer gelatins (sika antler gelatin, sika deer hide gelatin, and sika deer bone gelatin) were prepared through hot-water extraction of the antlers, hides, or bones from sika deer (*Cervus nippon*) inhabiting Hokkaido, Japan. The donkey hide gelatin was provided by Wakan SINCA Co., Ltd. (Tokyo, Japan). Bovine gelatin, porcine gelatin, and fish gelatin were purchased from Nippi, Inc. (Tokyo, Japan).

### 3.2. Sample Preparations

The powdered gelatin from each animal source was dissolved in 1.0 *w*/*v*% ammonium bicarbonate solution at a concentration of 2.0 mg/mL. Then, 100 mL of porcine trypsin solution (1.0 mg/mL) was added, and enzymatic digestion was performed at 37 °C for 12 h. The resulting peptide mixture solution was subsequently used for LC-MS/MS analysis.

### 3.3. LC-MS/MS Analysis

Ultra-high-performance liquid chromatography (UHPLC) was performed using a Vanquish UHPLC system (Thermo Scientific, Waltham, MA, USA). ODS chromatographic separation was carried out on a TSKgel ODS-120H (2.0 mm I.D. × 100 mm, 1.9 mm). The mobile phase consisted of A (0.1 vol% formic acid in distilled water) and B (0.1% formic acid in acetonitrile), with a gradient elution as follows: 0–15 min at 5–20% B and 15–20 min at 20–50% B. The mobile phase flow rate was maintained at 0.4 mL/min, and the column temperature was kept constant at 40 °C using a column temperature oven.

Mass spectrometry detection was performed using a Q-Exactive hybrid quadrupole-Orbitrap mass spectrometer (Thermo Scientific, Waltham, MA, USA) equipped with an ESI source, operated in positive ion mode. Data were acquired in full MS and full MS/data-dependent (dd)-MS/MS modes. The scan range was set at *m/z* 200 to 3000, and dd-MS/MS scans were performed using high-energy collision dissociation (HCD) with a stepped normalized collision energy (NCE) of 20, 40, and 60 eV. The in-source CID was set to 0 eV. The source parameters were set as follows: spray voltage, +3.5 kV; capillary temperature, 262.5 °C, sheath gas flow rate, 50 units; AUX gas flow rate, 12.5 units; sweep gas flow rate, 2.5 units; S-lens RF level, 50 units; probe heater temperature, 425 °C; resolution, 70,000 (for Full MS) or 35,000 (for full MS/dd-MS/MS); AGC target, 1 × 10^6^ (for full MS) or 1 × 10^5^ (for dd-MS/MS).

### 3.4. Data Processing and De Novo Sequencing Identification of Peptides

All mass spectrometry data were acquired using Thermo Scientific Xcalibur 4.1 software (Thermo Fisher Scientific, Waltham, MA, USA), and data processing was performed with Thermo Scientific FreeStyle 1.6 software (Thermo Fisher Scientific, Waltham, MA, USA). The extracted ion spectrum was generated by extracting the following mass ranges at 10.0 ppm mass tolerance: *m/z* 780.9101 [M + 2H]^2+^ (P11), *m/z* 732.8339 [M + 2H]^2+^ (R2), *m/z* 598.8227 [M + 2H]^2+^ (R3), *m/z* 775.8814 [M + 2H]^2+^ (R4), and *m/z* 737.7062 [M + 3H]^3+^ (trypsin-derived peptide).

Peak lists obtained from MS/MS spectra were identified using OMSSA version 2.1.9 [40] and X!Tandem version X! Tandem Vengeance (2015.12.15.2) [41]. The search was conducted using SearchGUI version 4.3.5 [42]. Protein identification was conducted against a concatenated target/decoy database [43] obtain from the UniProtKB database [44] considering the following species: bovine (*Bos taurus*), wild boar (*Sus scrofa*), horse (*Equus caballus*), donkey (*Equus asinus*), goat (*Capra hircus*), sheep (*Ovis aries*), European red deer (*Cervus elaphus hippelaphus*), white-tailed deer (*Odocoileus virginianu*), red deer (*Cervus elaphus*), Siberian musk deer (*Moschus moschiferus*), fallow deer (*Dama dama*), European roe deer (*Capreolus capreolus*), southern red muntjac (*Muntiacus muntjac*), and fish. The decoy sequences were created by reversing the target sequences in SearchGUI. The identification settings were as follows: trypsin, specific, with a maximum of 2 missed cleavages, 10.0 ppm as MS1 and 0.02 Da as MS2 tolerances. Peptides and proteins were inferred from the spectrum identification results using PeptideShaker version 3.0.8 [39].

## 4. Conclusions

The present study identified and validated characteristic marker peptide combinations that reliably distinguish sika deer gelatin from other animal-derived gelatins using LC-MS/MS analysis. By analyzing high-resolution mass spectrometry data from trypsin-digested peptide mixtures of gelatin samples and sequence information from public databases, the peptides P11 (GETGPAGPAGPIGPVGAR), R2 (SGETGASGPP(+15.99)GFAGEK), R3 (TGQP(+15.99)GAVGPAGIR), and R4 (GPP(+15.99)GESGAAGPAGPIGSR) were selected as species-specific marker peptides with high detection intensities in gelatin from sika deer antlers, hides, and bones. Notably, the marker peptide P11 has not been reported in other species of deer [32,33,34,35,36,37]. Comparative analyses with commercially available gelatins confirmed the exclusivity of these marker peptides to sika deer, with no significant detection in gelatins derived from donkey hide, bovine, porcine, or fish sources. Among the selected markers, the peptide P11 exhibited consistently high intensity across all sika deer gelatin samples, making it a particularly robust indicator. In addition, the peptides R2, R3, and R4 demonstrated distinctive patterns linked to the anatomical origin of the sika deer gelatin, suggesting their potential for further refinement in differentiating among sika deer gelatin types. In contrast, gelatins from other species presented distinct peptide profiles, underscoring the specificity of the marker peptide set for sika deer. The established marker peptides provide a reliable and efficient tool for verifying the authenticity of sika deer gelatin, thereby supporting its safe and effective use as a health-promoting resource.

## Figures and Tables

**Table 1:** Amino acid sequences of 28 marker peptide candidates (positive ion mode).

No.	t*_R_* (min)	*m/z*	Charge	Sequence ^a^	Chain
P1	2.34	418.7223	2	GPAGPQGPR	COL1A1
P2	2.48	434.7354	2	GPSGPQGIR	COL1A2
P3	4.48	392.2216	2	GAAGLPGPK(+15.99)	COL1A1
P4	4.55	666.8308	2	GPSGPQGPSGPP(+15.99)GPK	COL1A1
P5	5.04	553.2911	2	GVQGPP(+15.99)GPAGPR	COL1A1
P6	6.20	446.7536	2	PGPIGPAGAR	COL1A2
P7	7.77	449.7589	2	GVVGLPGQR(+15.99)	COL1A1
P8	7.91	730.3499	2	GAAGP(+15.99)P(+15.99)GATGFPGAAGR(+15.99)	COL1A1
P9	8.40	590.8253	2	TGQPGAVGPAGIR	COL1A2
P10	10.71	793.8815	2	GANG(+0.98)AP(+15.99)GIAGAPGFP(+15.99)GAR(+15.99)	COL1A1
P11	11.55	780.9101	2	GETGPAGPAGPIGPVGAR	COL1A1
P12	11.90	908.9360	2	GPPGP(+15.99)MGPPGLAGPPGESGR	COL1A1
R1	6.35	739.3578	3	GETGPAGRP(+15.99)GEVGPP(+15.99)GPP(+15.99)GPAGEK	COL1A1
R2	7.09	732.8339	2	SGETGASGPP(+15.99)GFAGEK	COL1A2
R3	7.39	598.8227	2	TGQP(+15.99)GAVGPAGIR	COL1A2
R4	7.45	775.8814	2	GPP(+15.99)GESGAAGPAGPIGSR	COL1A2
R5	8.32	644.3202	2	GFP(+15.99)GSP(+15.99)GNIGPAGK	COL1A2
R6	8.57	894.9656	2	AGPVGTAGAP(+15.99)GPQGPVGPTGK	COL1A2
R7	8.59	556.7801	2	GLAGPP(+15.99)GMP(+15.99)GAR	COL1A1
R8	10.15	536.7750	2	GFP(+15.99)GADGVAGPK	COL1A1
R9	10.39	1147.5565	2	GYP(+15.99)GNAGPVGTAGAP(+15.99)GPQGPVGPTGK	COL1A2
R10	10.39	765.3729	3	GYP(+15.99)GN(+0.98)AGPVGTAGAP(+15.99)GPQGPVGPTGK	COL1A2
R11	12.25	1275.1007	2	GNDGATGAAGPP(+15.99)GPTGPAGPP(+15.99)GFP(+15.99)GAVGAK	COL1A2
R12	14.57	611.8090	2	GFP(+15.99)GTP(+15.99)GLP(+15.99)GFK	COL1A2
R13	15.51	863.7582	3	GSDGSVGPVGPAGPIGSAGPP(+15.99)GFP(+15.99)GAP(+15.99)GPK	COL1A2
R14	16.32	858.4260	3	GSDGSVGPVGPAGPIGSAGPP(+15.99)GFPGAP(+15.99)GPK	COL1A2
R15	16.50	637.3487	2	LGAP(+15.99)GFLGLP(+15.99)GSR	COL1A2

^a^ P(+15.99): oxidation on Pro, K(+15.99): oxidation on Lys, R(+15.99): oxidation on Arg, G(+0.98): deamidation on Gly, N(+0.98): deamidation on Asn.

**Table 2:** Comparative analyses of sika deer gelatin with commercially available gelatins and BLAST search results.

No.	Peak Area Ratio Relative to the Trypsin-Derived Peptide (*m*/*z* 737.7062)							BLAST Search Results ^a^		
	Sika Deer Antler	Sika Deer Hide	Sika Deer Bone	Donkey Hide	Bovine	Porcine	Fish	Horse	Sheep	Goat
P1	4.99	4.94	3.76	6.43	4.15	3.50	0.05	✓	✓	✓
P2	3.90	3.07	2.73	4.38	3.69	4.77	2.88	-	✓	✓
P3	6.68	5.56	3.70	7.29	8.13	9.08	0.06	✓	✓	✓
P4	2.09	3.21	1.30	2.53	2.50	1.53	0.07	-	-	-
P5	6.81	5.45	4.94	7.32	5.85	4.40	0.05	✓	✓	✓
P6	3.90	4.37	2.37	5.85	5.37	4.63	0.05	✓	-	✓
P7	9.88	6.84	4.67	9.82	1.80	1.16	0.05	✓	✓	-
P8	3.54	3.31	2.60	3.82	4.94	5.62	0.04	-	-	-
P9	1.32	2.33	0.76	0.02	0.02	0.54	0.02	-	✓	✓
P10	3.47	2.13	2.31	2.71	4.61	5.58	0.03	-	✓	✓
P11	5.69	4.91	3.96	0.01	7.71	0.48	0.03	-	✓	✓
P12	1.70	2.37	1.25	0.01	0.58	0.76	0.89	-	-	-
R1	0.15	0.16	0.09	0.00	0.14	0.01	0.00	-	-	-
R2	1.05	0.89	0.66	0.01	0.00	0.00	0.00	-	-	-
R3	1.91	0.83	1.44	0.00	0.00	0.29	0.00	-	✓	✓
R4	2.59	1.99	1.35	2.24	0.03	3.32	0.07	✓	-	-
R5	0.91	1.25	0.74	0.95	0.04	0.00	0.02	✓	✓	✓
R6	0.24	0.28	0.19	0.00	0.00	0.00	0.00	-	-	-
R7	0.04	2.17	0.16	2.48	0.22	0.04	0.01	-	-	-
R8	0.00	0.32	0.00	0.05	0.11	0.02	0.00	✓	-	-
R9	0.32	0.26	0.23	0.00	0.00	0.00	0.00	-	-	-
R10	0.44	0.40	0.35	0.00	0.01	0.00	0.00	-	-	-
R11	0.15	0.03	0.05	0.05	0.19	0.41	0.00	-	-	-
R12	0.38	0.01	0.09	0.03	0.07	0.32	0.00	✓	✓	-
R13	0.08	0.00	0.03	0.01	0.03	0.01	0.00	✓	✓	✓
R14	0.04	0.02	0.01	0.01	0.02	0.00	0.00	✓	✓	✓
R15	0.13	0.16	0.09	0.00	0.23	0.01	0.00	-	✓	-
R16	0.10	0.09	0.04	0.00	0.00	0.00	0.00	-	✓	✓

^a^ COL1A1 and COL1A2 sequences for horse (UniProt Accession No. A0A5F5Q281 and F6RTI8), sheep (UniProt Accession No. W5P481 and W5NTT7), and goat (UniProt Accession No. A0AAJ7KZE5 and A0A452G3V6) were used as query sequences. “✓” indicates the presence of an identical sequence, while “-” indicates the absence of a matching sequence.

## Data Availability

All new research data were presented in this contribution.

## Supplementary Materials

The following supporting information can be downloaded at https://www.mdpi.com/article/10.3390/molecules30071528/s1, Figure S1: Total ion chromatograms (TIC) in positive ion mode of sika deer gelatin and commercially available gelatins; Figures S2−S4: Overlay of total ion chromatograms (TIC) in positive ion mode for triplicate digestion experiments of sika deer gelatin; Figures S5−S7: Extracted ion chromatograms (XIC) of marker peptides in three different lots of sika deer gelatin; Figures S8–S11: Mass and product ion spectrum for marker peptides P11, R2, R3, and R4; Table S1: Identified peptides in sika deer antler gelatin; Table S2: Identified peptides in sika deer bone gelatin; Table S3: Identified peptides in sika deer skin gelatin.
