# Supplementary material for: Identification of Marker Peptides in Gelatins from Sika Deer (Cervus nippon) Using Ultra-High-Performance Liquid Chromatography–Quadrupole-Exactive-Orbitrap Mass Spectrometry
Source: Molecules. 2025 Mar 29;30(7):1528. doi: 10.3390/molecules30071528 (PMC11990231; doi:10.3390/molecules30071528)
Supplement: Supplementary file 1 [file molecules-30-01528-s001.zip › FigureSI_R1_20250321.pdf]

Supporting Information

Identification of Marker Peptides in Gelatins from Sika Deer (*Cervus nippon*) Using Ultra-High-Performance Liquid Chromatography–Quadrupole-Exactive-Orbitrap Mass Spectrometry

Kouharu Otsuki [1,*], *Aya Nomizo* [1], *Mi Zhang* [1], *Dongxia Li* [2], *Takashi Kikuchi* [1], *Wei Li* [1,*]

1 Faculty of Pharmaceutical Sciences, Toho University, Miyama 2-2-1, Funabashi, Chiba 274-8510, Japan

2 Department of Medical Laboratory, Medical College of Dalian University, Dalian, Liaoning 116622, China

Contents

Figure S1. Total ion chromatograms (TIC) in positive ion mode of sika deer gelatin and commercially available gelatins S2

Figure S2. Overlay of total ion chromatograms (TIC) in positive ion mode for triplicate digestion experiments of sika deer antler gelatin S3

Figure S3. Overlay of total ion chromatograms (TIC) in positive ion mode for triplicate digestion experiments of sika deer hide gelatin S4

Figure S4. Overlay of total ion chromatograms (TIC) in positive ion mode for triplicate digestion experiments of sika deer bone gelatin S5

Figure S5. Extracted ion chromatograms (XIC) of maker peptides in three different lots of sika deer antler gelatin S6

Figure S6. Extracted ion chromatograms (XIC) of maker peptides in three different lots of sika deer hide gelatin S7

Figure S7. Extracted ion chromatograms (XIC) of maker peptides in three different lots of sika deer bone gelatin S8

Figure S8. Mass and product ion spectrum for maker peptide P11 .... S9

Figure S9. Mass and product ion spectrum for maker peptide R2 .... S10

Figure S10. Mass and product ion spectrum for maker peptide R3 .... S11

Figure S11. Mass and product ion spectrum for maker peptide R4 .... S12

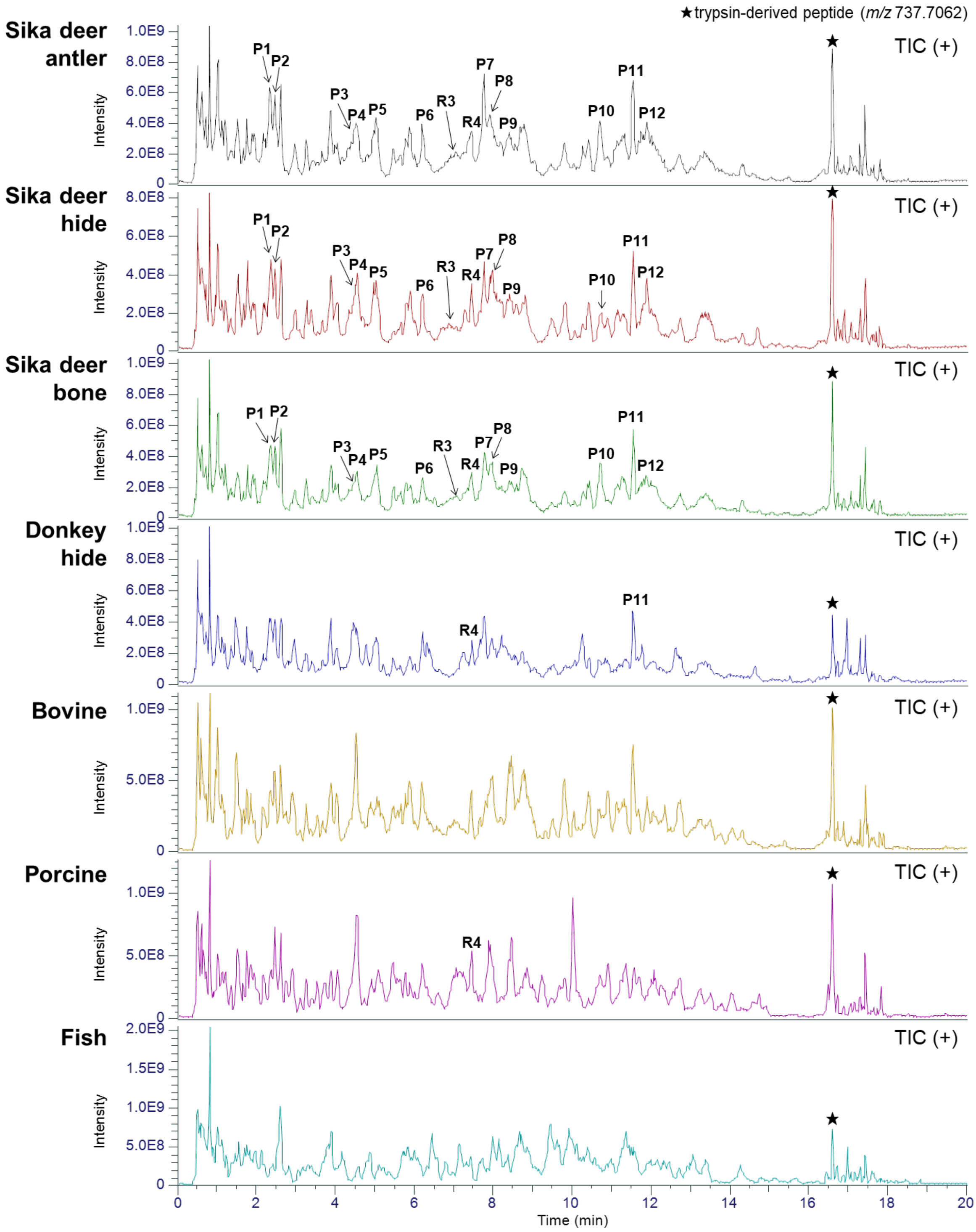

Figure S1. Total ion chromatograms (TIC) in positive ion mode of sika deer gelatin and commercially available gelatins.

Sika deer antler

Lot: Antler20250208

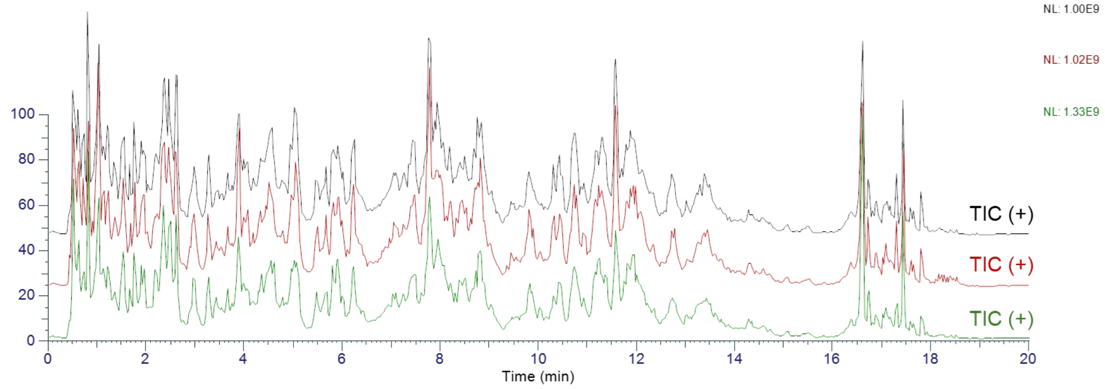

Lot: Antler20231114

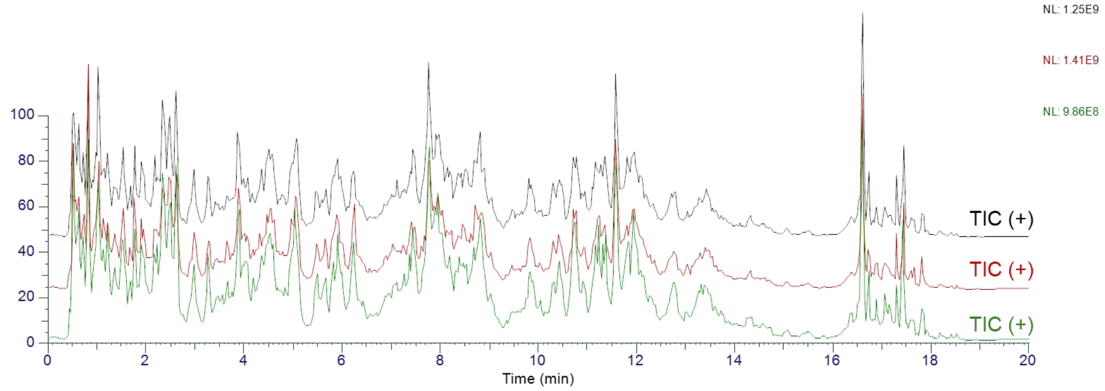

Lot: Antler20210310

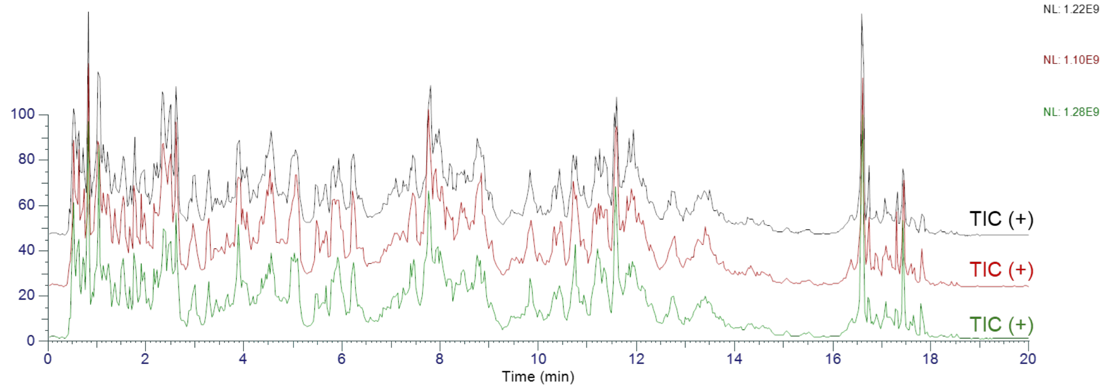

Figure S2. Overlay of total ion chromatograms (TIC) in positive ion mode for triplicate digestion experiments of sika deer antler gelatin.

Sika deer hide

Lot: Hide20250208

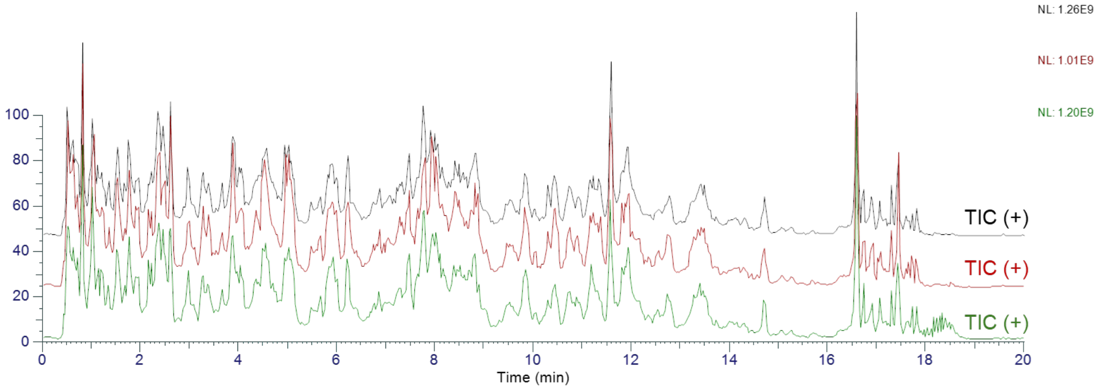

Lot: Hide20240411

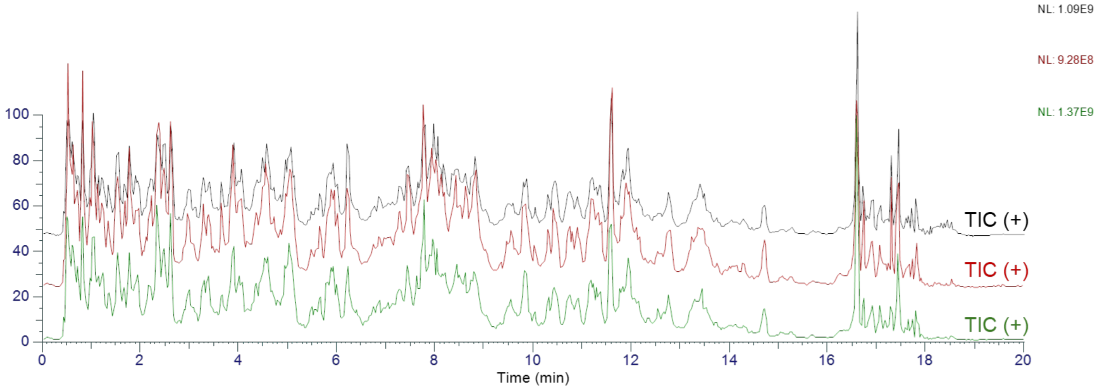

Lot: Hide20210316

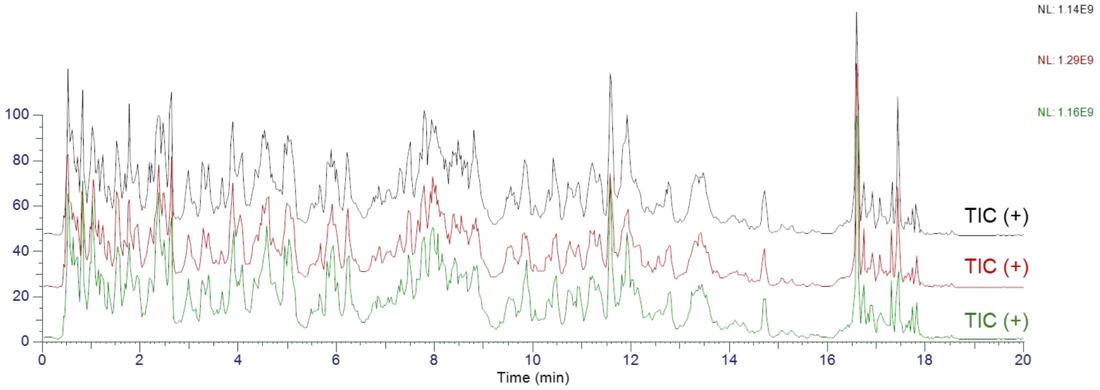

Figure S3. Overlay of total ion chromatograms (TIC) in positive ion mode for triplicate digestion experiments of sika deer hide gelatin..

Sika deer hide

Lot: Bone20250208

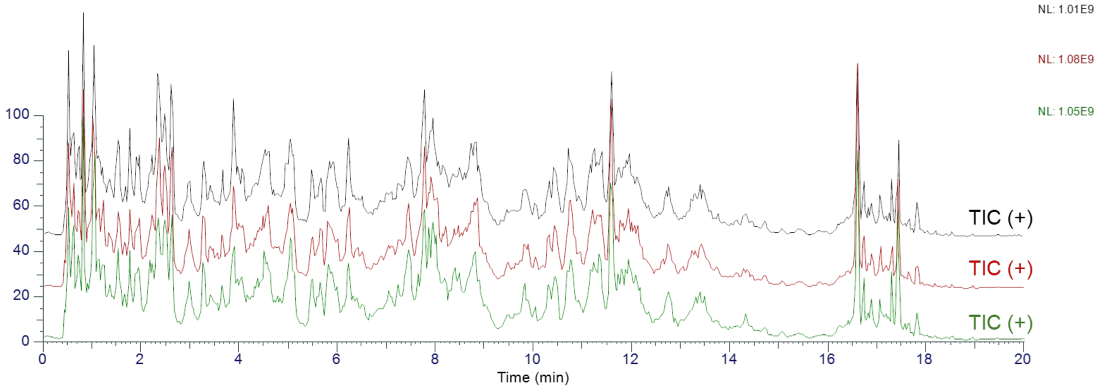

Lot: Bone20231114

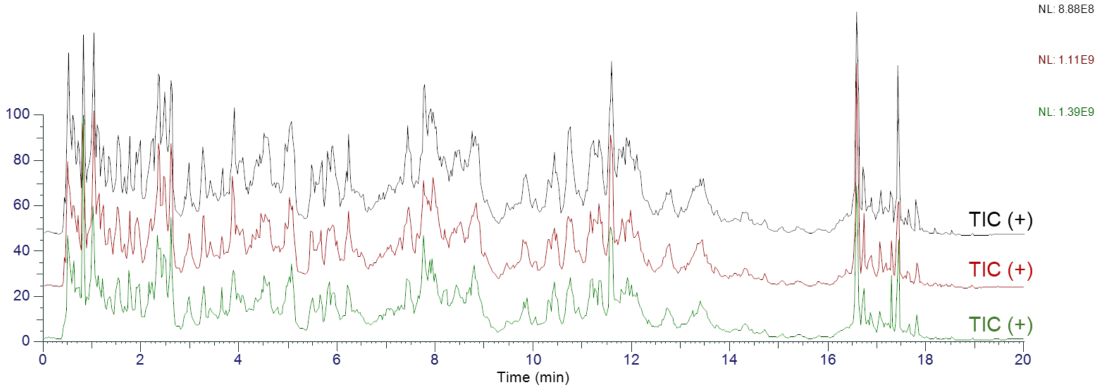

Lot: Bone20210316

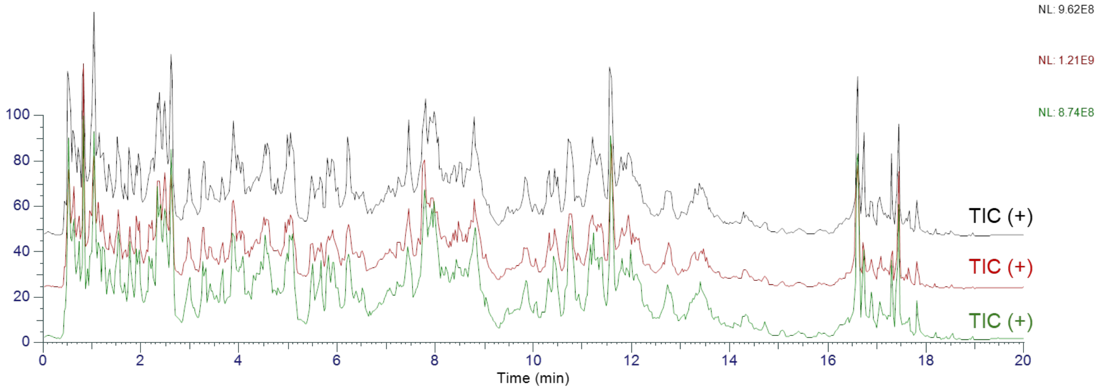

Figure S4. Overlay of total ion chromatograms (TIC) in positive ion mode for triplicate digestion experiments of sika deer bone gelatin..

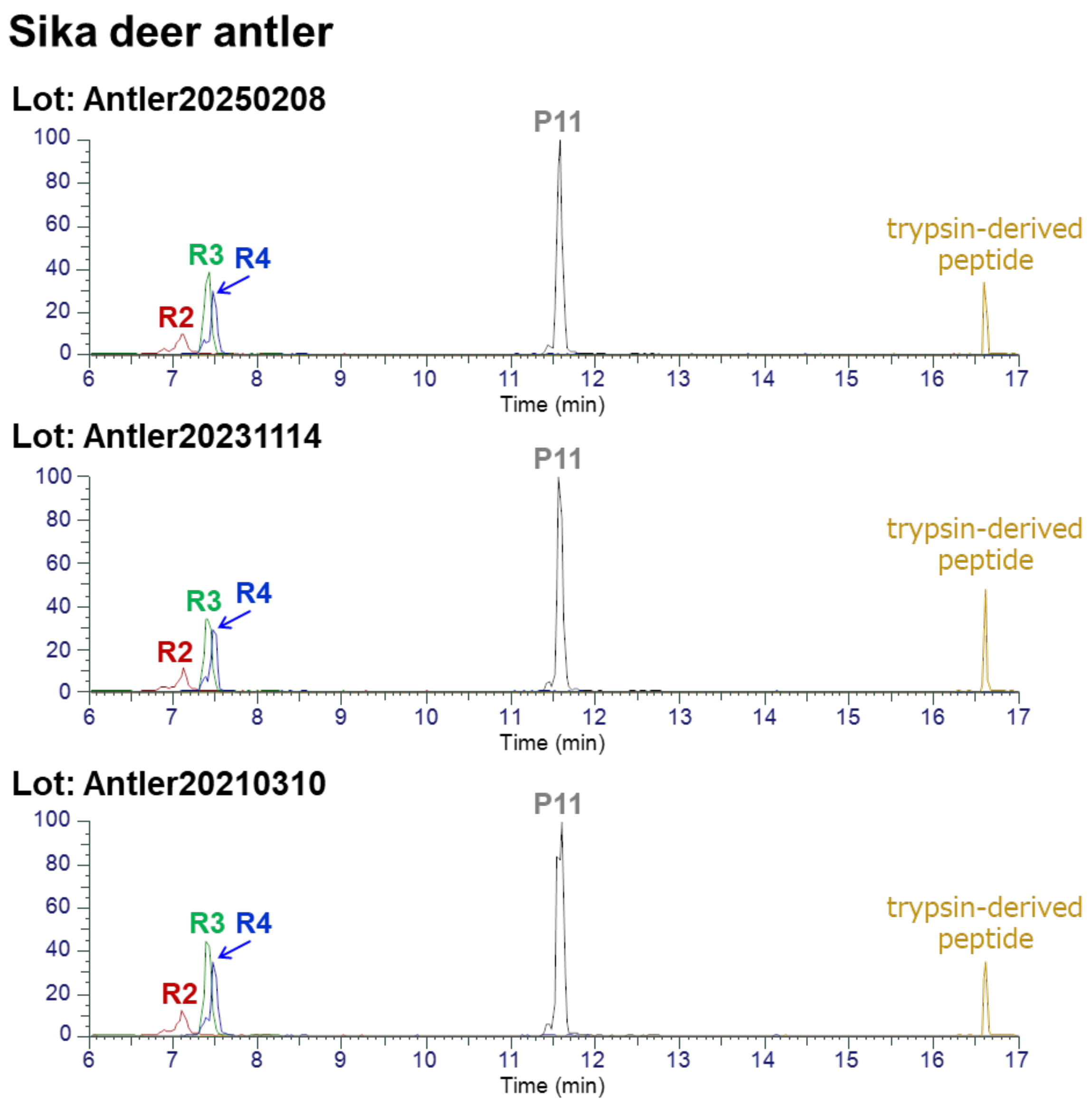

Figure S5. Extracted ion chromatograms (XIC) of maker peptides in three different lots of sika deer antler gelatin.

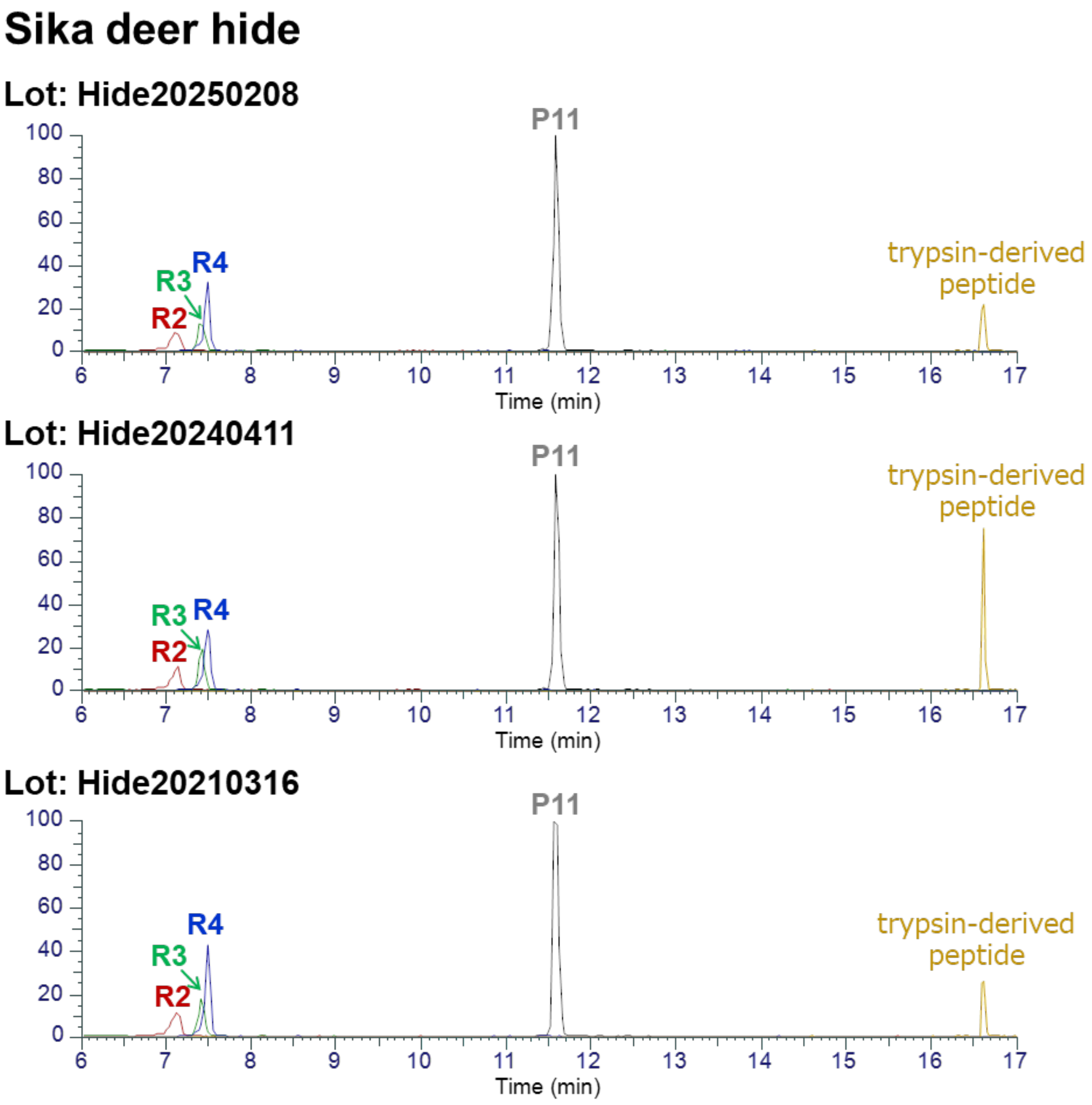

Figure S6. Extracted ion chromatograms (XIC) of maker peptides in three different lots of sika deer hide gelatin.

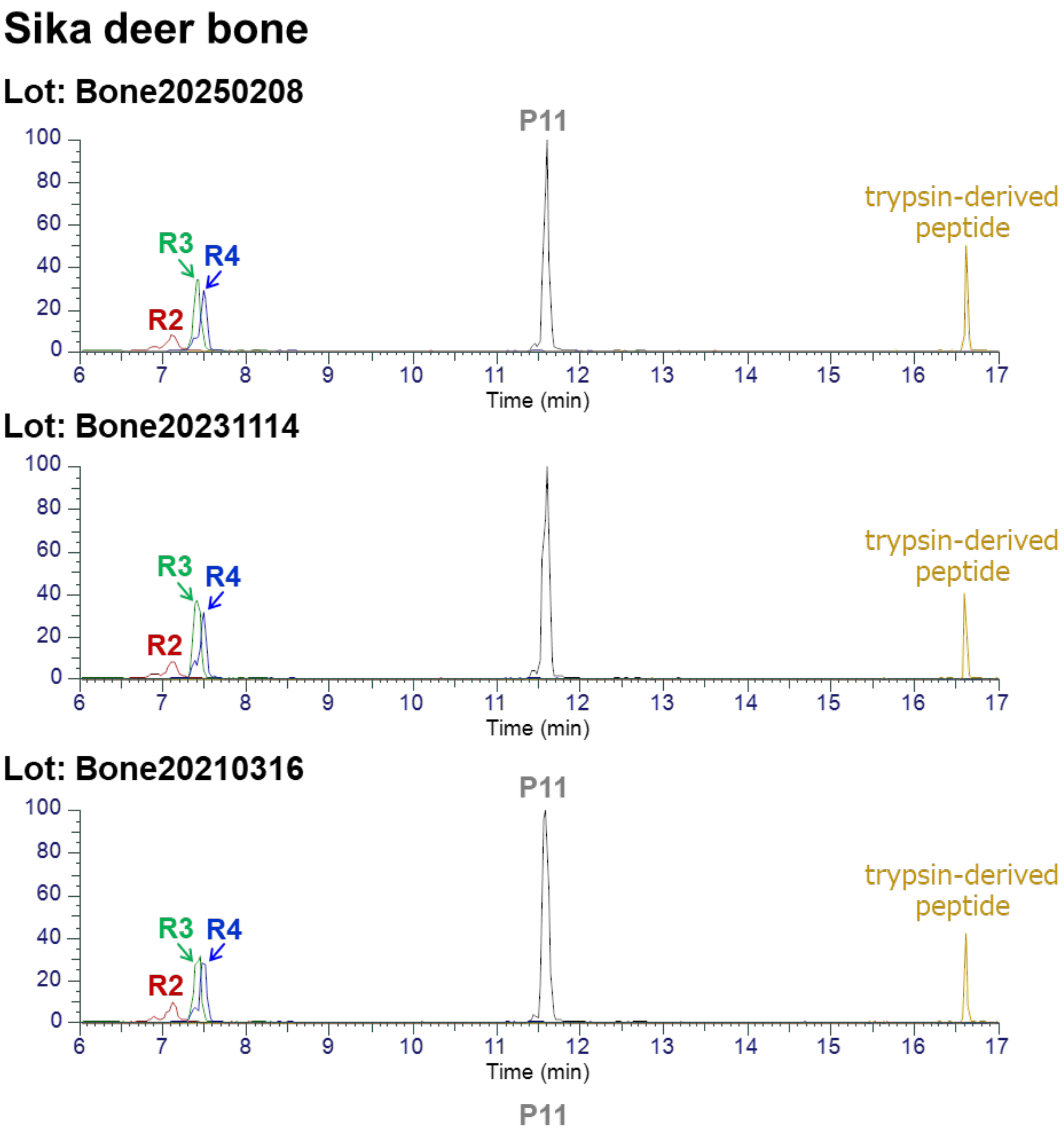

Figure S7. Extracted ion chromatograms (XIC) of maker peptides in three different lots of sika deer bone gelatin.

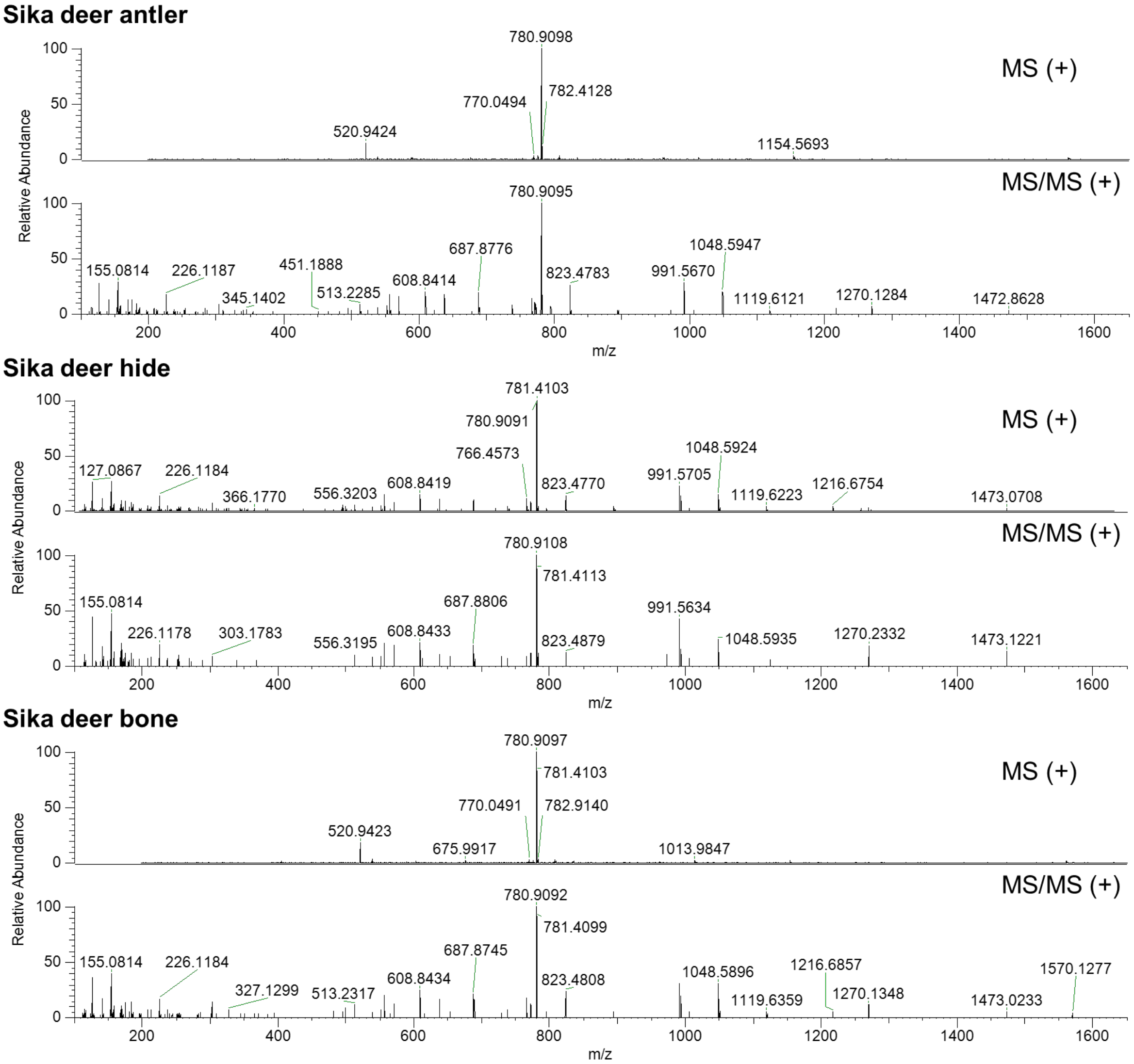

Figure S8. Mass and product ion spectrum for maker peptide P11.

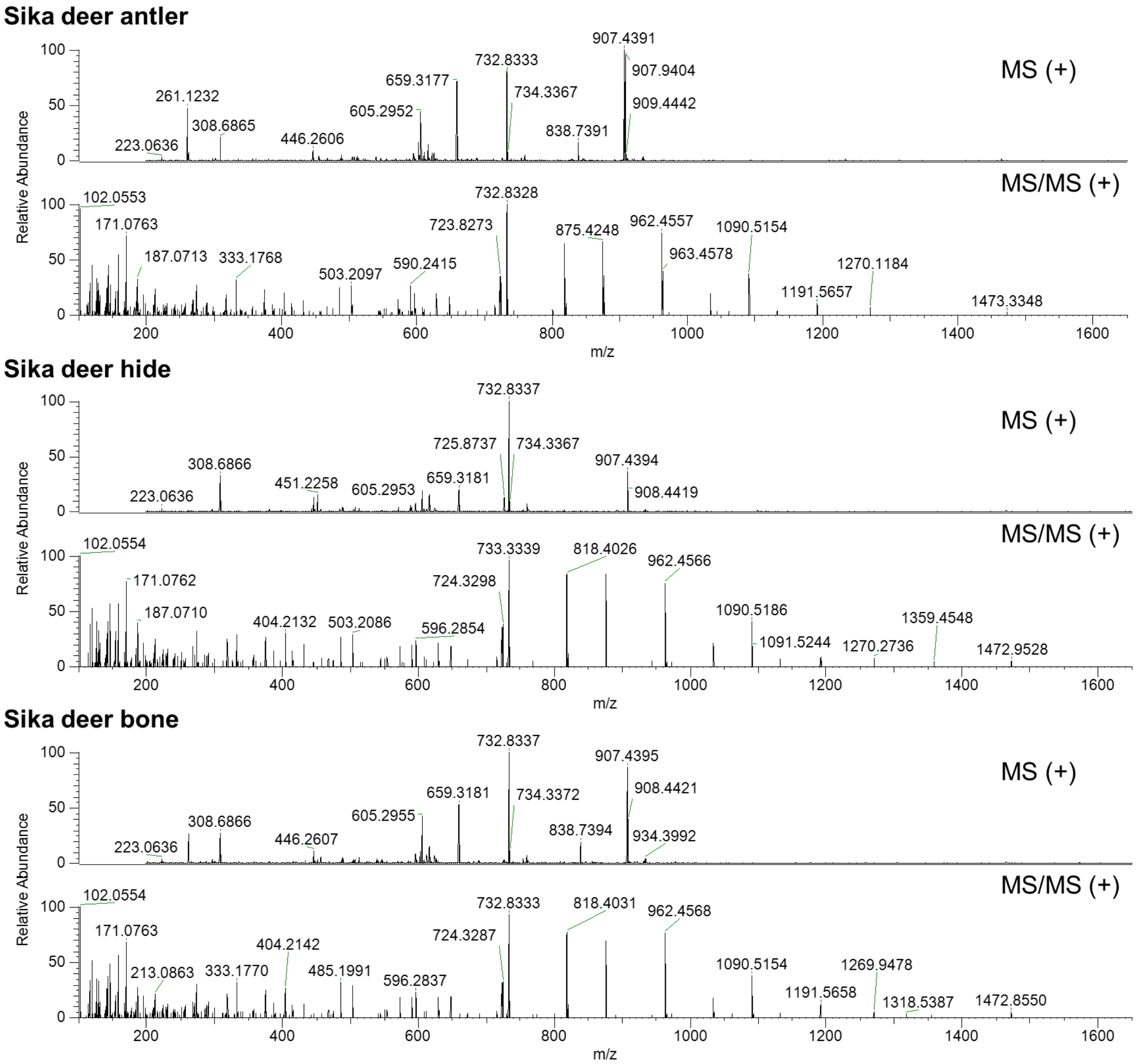

Figure S9. Mass and product ion spectrum for maker peptide R2.

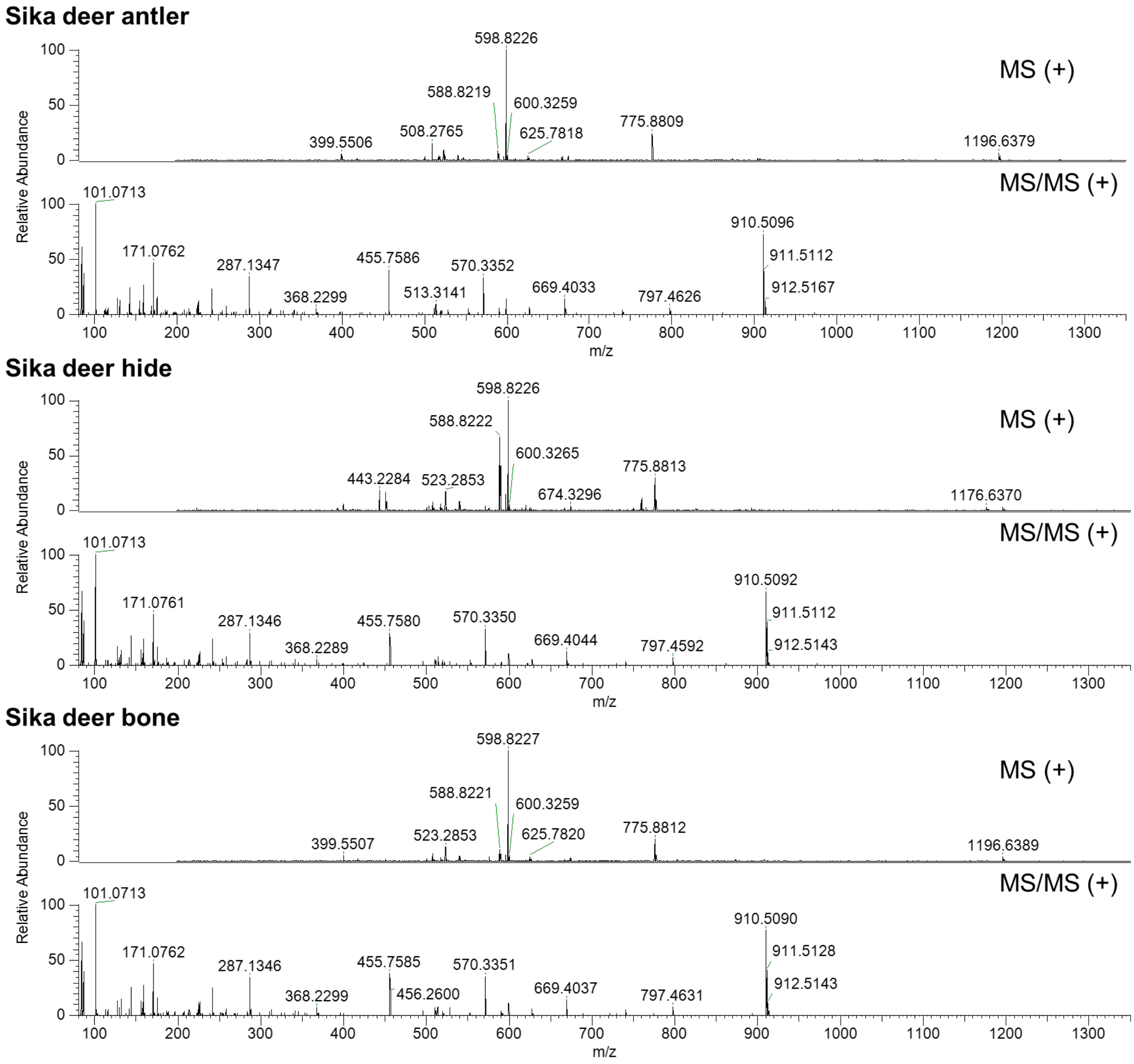

Figure S10. Mass and product ion spectrum for maker peptide R3.

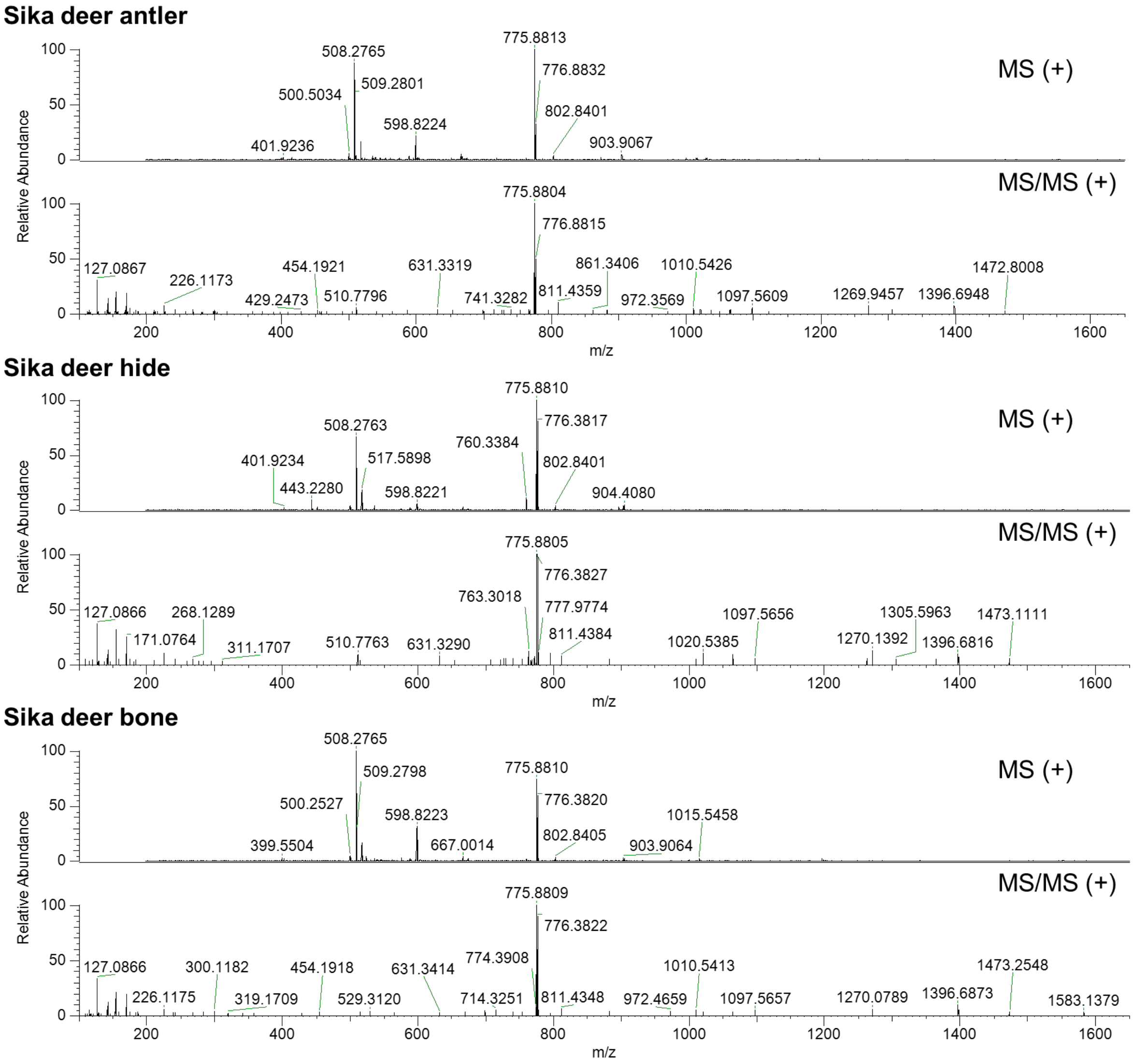

Figure S11. Mass and product ion spectrum for maker peptide R4.
